# An Isoxazole Derivative SHU00238 Suppresses Colorectal Cancer Growth through miRNAs Regulation

**DOI:** 10.3390/molecules24122335

**Published:** 2019-06-25

**Authors:** Haoyu Wang, Yurui Ma, Yifan Lin, Jiajie Liu, Rui Chen, Bin Xu, Yajun Liang

**Affiliations:** 1 Department of Chemistry, Qianweichang College, Shanghai University, Shanghai 200444, China; hughie@shu.edu.cn (H.W.); miaomiao3327@126.com (Y.L.); ejay030201@163.com (J.L.); 2 School of Life Science, Shanghai University, Shanghai 200444, China; Myr819374294@163.com (Y.M.); rchen94@hotmail.com (R.C.); 3 Innovative Drug Research Center, Shanghai University, Shanghai 200444, China

**Keywords:** colorectal cancer, isoxazole derivative, miRNAs, SHU00238, drug discovery

## Abstract

Colorectal cancer (CRC) is a leading cause of cancer-related deaths worldwide. Isoxazoline and isoxazole derivatives represent an important class of five-membered heterocycles, which play a pivotal role in drug discovery. In our previous study, we developed a series of isoxazole derivatives with an efficient method. In this study, we evaluated their effects on tumor cell growth. HCT116 cells were treated with isoxazole derivatives; an cholecystokinin octapeptide (CCK-8) assay was used to calculate the IC_50_ (half maximal inhibitory concentration) of each derivative. Compound SHU00238, which was obtained by the copper nitrate-mediated [2+2+1] cycloaddition reaction of olefinic azlactone with naphthalene-1,4-dione, has a lower IC_50_; we analyzed its inhibitory activity in further assays. Cell apoptosis was estimated by flow cytometry analysis in vitro. SHU00238 injection was used to treat tumor-bearing mice. We found that SHU00238 suppressed cell viability and promoted cell apoptosis in vitro. SHU00238 treatment significantly inhibited colonic tumor growth in vivo. Furthermore, we compared the miRNAs expression changes in HCT116 cells before and after SHU00238 treatment. MiRNA profiling revealed that SHU00238 treatment affected cell fate by regulating a set of miRNAs. In conclusion, SHU00238 impedes CRC tumor cell proliferation and promotes cell apoptosis by miRNAs regulation.

## 1. Introduction

Colorectal cancer (CRC) is a leading cause of cancer-related deaths worldwide [1]. Genetic and epigenetic alterations lead to the initiation of colorectal cancer. In addition, colorectal cancer is closely related with lifestyle, age, and inflammatory disease [2,3,4,5,6]. Most cases of colorectal cancer are sporadic and slowly develop through adenoma-carcinoma sequences within a few years [7]. The five-year relative survival rate is greater than 90% in stage I patients. However, the survival rate is slightly above 10% in stage IV patients [8,9,10]. There is a critical need for the investigation of novel, molecular-targeted therapeutics for CRC [11].

Isoxazole is a member of five-membered heterocycle; its adjacent position is composed of two heteroatoms, an oxygen atom and a nitrogen atom [12]. The featured structure makes it easy to form non-covalent interaction. Isoxazole compounds have a wide range of biological activities, including anti-cancer, anti-bacterial, anti-fungal, anti-viral, and anti-bacterial [13,14,15]. For instance, sulfisoxazole has been applied in urinary tract infections and risperidone has been approved to treat schizophrenia in adults [16]. In our previous study, we developed a novel, copper nitrate-mediated [2+2+1] cycloaddition reaction and provided an alternative route for the expedient synthesis of pharmacologically interesting 3-aryl substituted isoxazolines and isoxazoles [17]. With this method, we synthesized a series of isoxazole derivatives. In the present study, we determined their anti-tumor activities.

MicroRNAs (miRNAs, miRs) are ~21–23-nucleotide, single-stranded RNAs that play tremendous regulatory roles in many biological processes; their dysregulation contributes to many diseases, including cancer [18,19,20]. MiRNAs regulate gene expression via binding to the 3′-untranslated region of genes [21,22]. Approximately two-thirds of protein-coding genes are regulated by miRNAs [23]. In this study, we synthesized 37 isoxazoline and isoxazole derivatives and determined their inhibitory activities in HCT116 cells. Among them, SHU00238 inhibited CRC cell viability more efficiently; Supplementary Material provides the preparation and spectroscopic data of SHU00238. In vitro and in vivo data revealed that SHU00238 suppressed CRC growth. Furthermore, we performed miRNA profiling with HCT116 cells treated with SHU00238. The data revealed that a set of miRNAs are significantly regulated by SHU00238, which regulated cell fate by targeting several cellular signaling pathways. Among the regulated miRNAs, miR-297, miR-30e-3p, miR-181d-5p, and miR-9-3p are known tumor regulators [24,25,26,27,28,29]. Therefore, the present study reveals that SHU00238 suppresses colorectal cancer growth through miRNAs regulation.

## 2. Results

### 2.1. An Isoxazole Derivative SHU00238 has a Lower IC_50_ in HCT116 Cells

**Figure 1:**
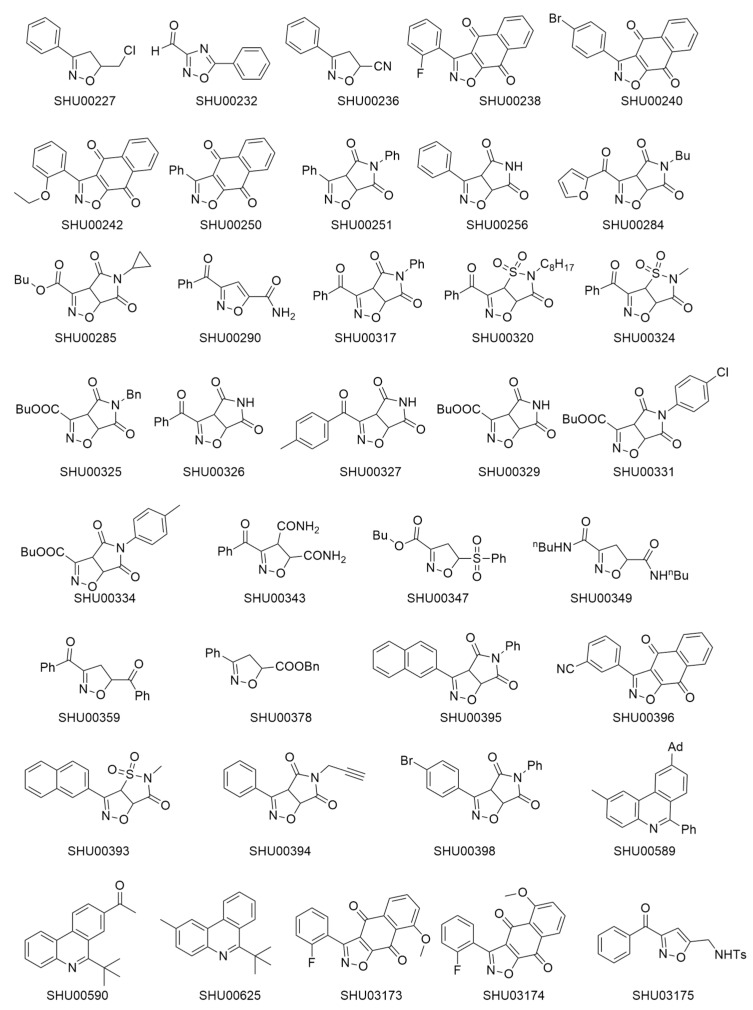
Chemical structure of 37 candidate compounds.

**Figure 2:**
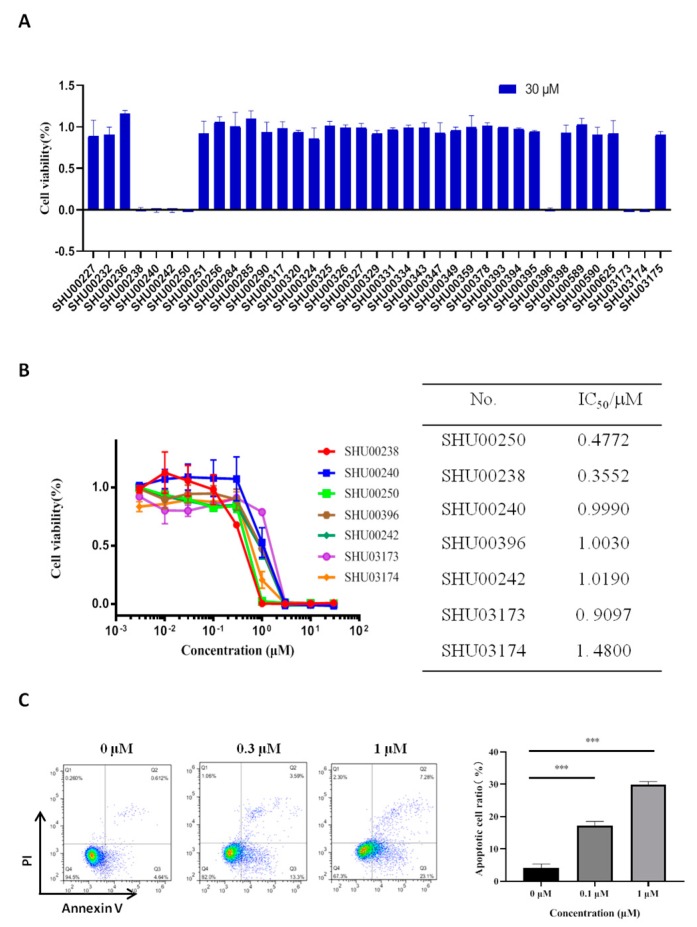
Seven compounds of 37 isoxazole derivatives suppress cell viability. (**A**) Cell viability analysis of HT116 cells treated with 30 μM isoxazole derivatives, as determined by CCK-8 assay. (**B**) The IC_50_ value of seven effective compounds in HCT116 cells, as determined by CCK-8 assay. (**C**) Apoptosis analysis of HCT116 cells treated with 0.3 μM, 1 μM SHU00238 (48 h, *n* = 3). ***, *p* < 0.001.

The chemical structure of 37 candidate compounds is seen in Figure 1. We examined their inhibitory activities in HCT116 cells. Preliminary data showed that SHU00238, SHU00240, SHU00242, SHU00250, SHU00396, SHU03173, and SHU03174 could prohibit colonic tumor cells growth, as seen in Figure 2A. Furthermore, we determined the IC_50_ of these compounds with CCK-8 assay. The results showed that SHU00238 inhibited HCT116 cell viability with a lower IC_50_ value of 0.3552 μM, as seen in Figure 2B. When we detected the cell apoptosis by AnnexV and PI (propidium iodide) staining, we found that SHU00238 treatment promoted cell apoptosis prominently, as seen in Figure 2C.

### 2.2. SHU00238 Suppresses Colonic Tumor Growth and Cell Proliferation in Xenograft Mice Model.

**Figure 3:**
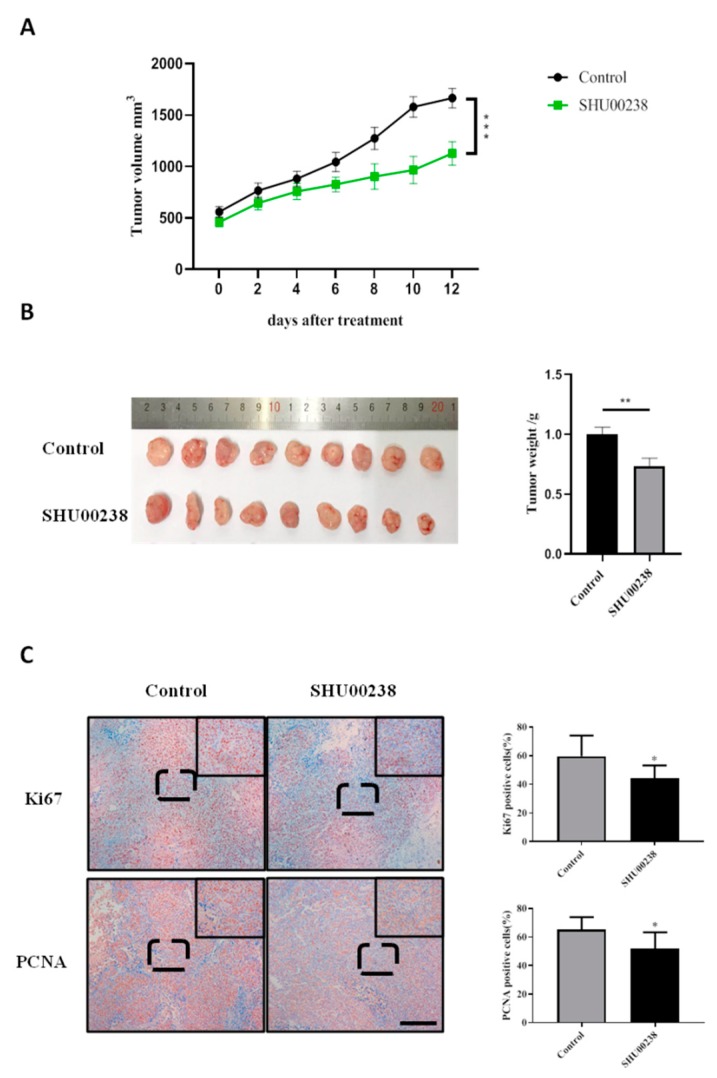
SHU00238 suppresses colonic tumor growth and cell proliferation in the xenograft mice model. (**A**) Tumor volume of control and SHU00238-treated mice (40 mg/kg, *n* = 9). (**B**) Tumors of control and SHU00238-treated mice at the end of experiment. (**C**) Ki67 and PCNA positive cells in tumor tissues. Scale bar, 200 μM, *, *p* < 0.05.

We explored the therapeutic effects of SHU00238 in tumor-bearing mice. HCT116 cells were injected subcutaneously in nude mice and SHU00238 administration was performed when the tumor size reached 500 mm^3^. Results showed that SHU00238 significantly reduced xenograft tumor volume, as seen in Figure 3A,B. In addition, we detected the expression of proliferation biomarkers in tumor tissues with immunohistochemical analysis. Ki67 and PCNA (Proliferating cell nuclear antigen) staining revealed that tumor cell proliferation was prominently reduced after SHU00238 treatment, as seen in Figure 3C.

### 2.3. SHU00238 Treatment Affects Cell Fate by Regulating a Set of miRNAs

**Figure 4:**
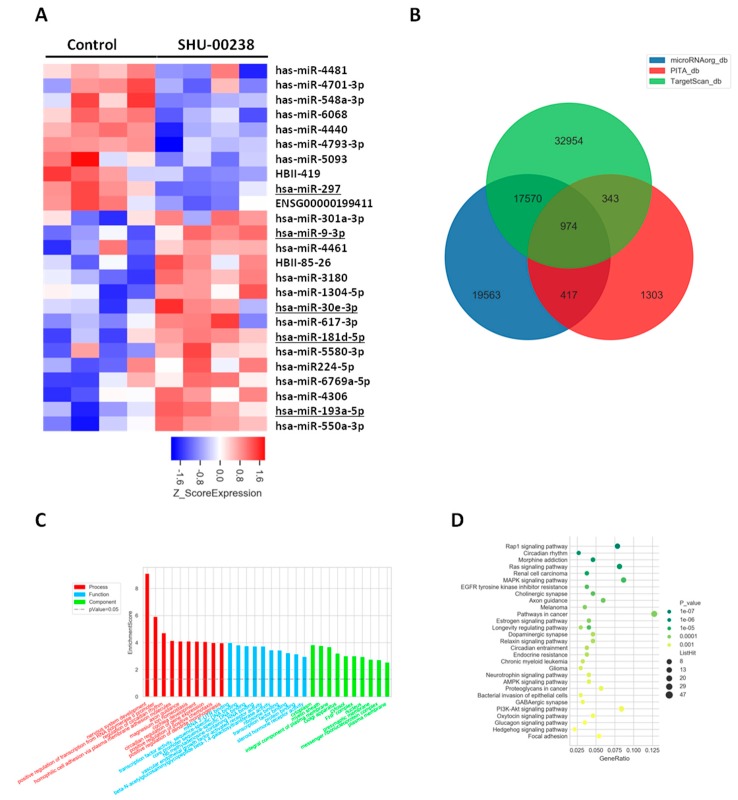
SHU00238 treatment affects cell fate by regulating a set of miRNAs. (**A**) Heatmap of the differential miRNAs. (**B**) Target genes of differentially expressed miRNAs from the intersection predicted with three databases (Targetscan, PITA, microRNAorg). (**C**) GO analysis and (**D**) KEGG analysis of the target genes. SHU00238 suppresses colonic tumor growth and cell proliferation in xenograft mice model.

To investigate the underlying mechanism of SHU00238 in tumor suppression, we performed miRNA microarray with HCT116 cells. A bioinformatics analysis revealed that SHU00238 treatment significantly changed miRNA expression. Among these miRNAs, the known tumor regulators were marked with underlines, as seen in Figure 4A. MiR-9-3p was identified as the tumor-suppressor miRNA and performed its functions by targeting TAZ expression in liver cancer [28]. MiR-181a-5p prevents cancer metastasis by targeting MMP-14 [27]. A blockade of miR-193a-5p increases the chemosensitivity of prostate cancer cells to docetaxel. MiR-30e-3p functions as a tumor suppressor through targeting Snail1 in clear cell renal cell carcinoma [29]. To explore the regulation of SHU00238 to cell fate, we analyzed the downstream genes of the regulated miRNAs, as seen in Figure 4B. GO(Gene Ontology) enrichment and KEGG(Kyoto Encyclopedia of Genes and Genomes) analysis revealed that cell fate-related signaling pathways were significantly changed by SHU00238 treatment, as seen in Figure 4C,D. Overall, our study demonstrates that SHU00238 suppresses colonic tumor growth in vitro and in vivo. SHU00238 affects cell fate by regulating a set of miRNAs.

## 3. Discussion

Isoxazole derivatives have a broad biological activities and play increasing important roles in drug discovery [13,30]. In our previous study, we developed an efficient route for the expedient synthesis of pharmacologically interesting 3-aryl substituted isoxazolines and isoxazoles [17]. In the present study, we determined their inhibitory activity in colonic cancer cells. Among them, SHU00238, SHU00240, SHU00242, SHU00250, SHU00396, SHU03173, and SHU03174, which all share a similarly featured structure, prevent tumor cell viability efficiently. The compound SHU00238 has a lower IC_50_ value in HCT116 cells, indicating its promising effects in tumor suppression. 

Further analysis demonstrated that SHU00238 treatment promotes cell apoptosis in vitro. When we treated tumor-bearing mice with SHU00238, tumor volume and colonic tumor cell proliferation significantly decreased. Taken together, SHU00238 can inhibit colonic tumor growth both in vitro and in vivo, which indicates therapeutic application in tumor therapy. However, cancer is a complex disease; extensive studies are needed to determine the safety and metabolism of SHU00238 in tumor suppression [31].

To investigate the underlying mechanism of SHU00238 in tumor suppression, miRNAs profiling was performed [32,33,34]. Several known tumor regulators are significantly regulated by SHU00238 treatment. The functions of the other miRNAs in tumor progression are still to be elucidated. GO enrichment and KEGG analysis revealed that SHU00238 might affect cell fate by regulating several signaling pathways, such as Rap1 signaling pathway, Ras signaling pathway, MAPK signaling pathway, AMPK signaling pathway, pathways in cancer, and PI3K-Akt signaling pathway. However, further studies are needed to explain the mechanisms of SHU00238 treatment in tumor progression. Target screen works still need to be elucidated because isoxazoles are facilitate to form non-covalent interaction with other molecules.

## 4. Materials and Methods 

### 4.1. Cell Culture

Colorectal cancer cell HCT116 is a human colon cancer cell line. HCT116 was bought from the Chinese Academy of Sciences Cell Bank. Cells was cultured in Dulbecco’s modified Eagle’s medium (DMEM, Corning, New York, NY, USA) with 10% fetal bovine serum (FBS, CellMax, Shanghai, China) and 1% penicillin-streptomycin (PS, Gibco) at 37 °C with 5% CO_2_.

### 4.2. Cell Viability Assay

Cell viability was detected with cholecystokini ocatapeptide (CCK-8) kit (Bioworld, Shanghai, China). HCT116 cells were plated in 96-well plate at 2 × 10^5^/mL; cells were then treated with gradient-diluted compounds for 24 h. Cells were incubated with CCK-8 at 37 °C for 1 h; we then measured the optical density values (OD) 450 with microplate reader (Bio-Rad, Hercules, CA, USA). The IC_50_ of each compound was calculated using SPSS software by logit-transformed probit model.

### 4.3. Cell Apoptosis Assay

AnnexinV and PI Assay Kit (Beyotime, Shanghai, China) was used to measure the apoptosis level of HCT116 cells. Briefly, HCT116 cells were treated with DMSO or compound. After 24 h, cells were resuspended in 100 μL PBS. Cells were stained with Annexin V and PI at 4 °C for 20 min and then these samples were detected with flow cytometry (Beckman, Brea, CA, USA).

### 4.4. Tissue Immunohistochemistry

Xenograft tumor tissues were embedded in paraffin and sliced into 5 μm sections. Samples were incubated in 65 °C for 2 h and then were dewaxed and dehydrated. Samples were boiled for 10 min to unmask antigens. Next, samples were immersed in 0.3% H_2_O_2_ for 10 min to destruct the endogenous peroxidase activity. We subsequently blocked the sections with 5% BSA, added 50 μL primary antibodies against Ki67 (Abcam, 1:300, *v*/*v*, dilution) or PCNA (Proteintech, 1:300, *v*/*v*, dilution), and incubated overnight at 4 °C. Secondary antibodies were added for 1 h at room temperature. Next, 3,3-diaminobenzidine tetrahydrochloride (DAB) solution was used to amplify the signals. A total of 15 fields were randomly selected for each section under the microscope.

### 4.5. Microarray Hybridization and Data Analysis

Two group of total RNA from HCT116 with or without SHU00238 treatment were subjected to hybridization to Affymetrix miRNA4.0 arrays, performed by Shanghai OE Biotechnology Corporation. The microarray data was normalized according to the median intensity of each sample. Differentially expressed miRNAs were then identified through fold change as well as *p*-value calculated using a t-test. The threshold set for up- and down-regulated genes was a fold change >= 2.0 and a *p*-value <= 0.05. The miRNA array data have been deposited in GEO database. The accession number is GSE 132619. 

### 4.6. Mice and Treatment

Athymic nude mice were purchased from the Cavens lab (Changzhou, China). All animal experiments were conducted according to institutional guidelines. Tumor-bearing mice were generated by injecting 3 × 10^6^ HCT116 cells subcutaneously in eight-week-old mice. Two weeks later, compound (50 mM, 1:5 dilution, 40 μL) was injected intraperitoneally every other day. Xenograft tumors were dissected after 14 days. Tumor samples were fixed in 4% PFA and stored in liquid nitrogen for later use. The protocol for this study was approved by the Animal Experiments Ethics Committee of School of Life Science in Shanghai University. All surgical procedures were performed under isoflurane anesthesia, and all efforts were made to minimize animal suffering and to reduce the number of animals used.

### 4.7. Statistical Analysis

All data were expressed as mean ± SED. All statistical analyses were performed through IBM (Armonk, NY, USA) SPSS Statistics 20 and variables between groups were subject to either independent sample; a t-test was used for comparisons between two groups. One-way analysis of variance (ANOVA) was used for multiple comparisons. *p*-Values less than 0.05 were considered statistically significant.

## Supplementary Materials

The following are available online.
