# Supplementary material for: An Isoxazole Derivative SHU00238 Suppresses Colorectal Cancer Growth through miRNAs Regulation
Source: Molecules. 2019 Jun 25;24(12):2335. doi: 10.3390/molecules24122335 (PMC6630644; doi:10.3390/molecules24122335)
Supplement: Supplementary file 1 [file molecules-24-02335-s001.pdf]

## Supplementary materials

*Preparation of Isoxazole Derivative SHU00238*

Isoxazole derivative SHU00238 was prepared according to the reported method [1]. The melting point was measured on a WRS-1A Digital Melting Point Apparatus without correction. Infrared spectrum was obtained using an AVATAR 370 FT-IR spectrometer. $^{1}$H, $^{13}$C and $^{19}$F-NMR spectra were recorded on a Bruker AV-500 spectrometer. Mass spectrum was measured on an Agilent 5975C using an electron impact technique.

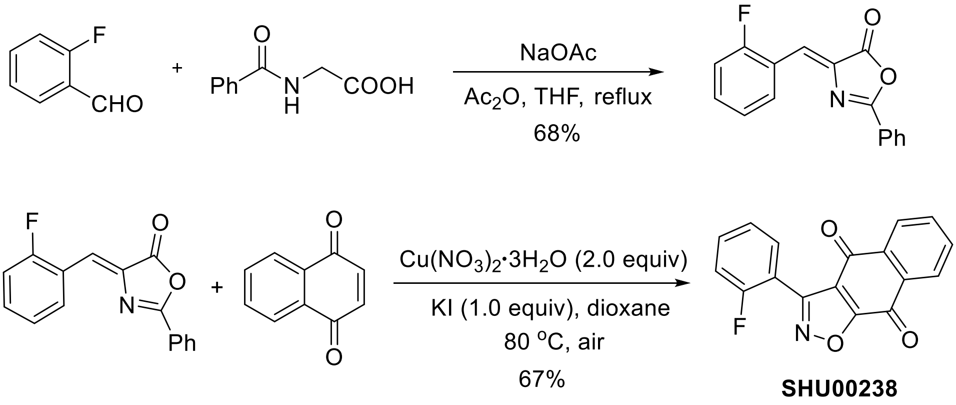


**Scheme 1**. Synthesis of isoxazole derivative SHU00238.

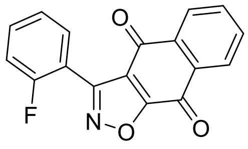

**SHU00238**

**3-(2-Fluorophenyl)naphtho[2,3-*d*]isoxazole-4,9-dione (SHU00238)** [1]**:** M.p. 192-194 °C; IR (KBr, $cm^{-1}$): 3077, 1686, 1584, 1514, 1465, 1336, 1197, 918, 758, 716; $^{1}$H NMR ($CDCl_3$, 500 MHz): $\delta$ 8.31-8.29 (m, 1H), 8.23-8.21 (m, 1H), 7.87-7.82 (m, 2H), 7.70 (td, $J$ = 7.4, 1.8 Hz, 1H), 7.62-7.57 (m, 1H), 7.35-7.27 (m, 2H); $^{19}$F NMR ($CDCl_3$, 470 MHz): $\delta$ -111.2 (m, Ar-F); $^{13}$C NMR ($CDCl_3$, 125 MHz): $\delta$ 178.1, 173.2, 165.5, 160.6 (d, $^{1}J_{C\text{-}F}$ = 252.8 Hz), 156.4, 135.3, 134.4, 133.6, 133.1 (d, $^{3}J_{C\text{-}F}$ = 8.4 Hz), 132.0, 131.2 (d, $^{4}J_{C\text{-}F}$ = 1.2 Hz), 127.5 (d, $^{3}J_{C\text{-}F}$ = 9.1 Hz), 124.4 (d, $^{4}J_{C\text{-}F}$ = 3.6 Hz), 120.6, 116.2 (d, $^{2}J_{C\text{-}F}$ = 21.0 Hz), 114.7 (d, $^{2}J_{C\text{-}F}$ = 14.4 Hz); EI-MS m/z: 293 [$M^{+}$].

## Reference


1. Lin, Y.; Zhang, K.; Gao, M.; Jiang, Z.; Liu, J.; Ma, Y.; Wang, H.; Tan, Q.; Xiao, J.; Xu, B. Copper nitrate-mediated synthesis of 3-aryl isoxazolines and isoxazoles from olefinic azlactones. *Org. Biomol. Chem.* **2019**, *17*, 5509-5513.
